# Biomass-Tuned Reduced Graphene Oxide@Zn/Cu: Benign Materials for the Cleanup of Selected Nonsteroidal Anti-inflammatory Drugs in Water

**DOI:** 10.1021/acsomega.2c07769

**Published:** 2023-02-14

**Authors:** Ajibola A. Bayode, Mercy T. Folorunso, Brigitte Helmreich, Martins O. Omorogie

**Affiliations:** † Department of Chemical Sciences, Faculty of Natural Sciences, Redeemer’s University, P.M.B. 230, 232101 Ede, Nigeria; ‡ Laboratório de Química Analítica Ambiental e Ecotoxicologia (LaQuAAE), Departamento de Química e Física Molecular, Instituto de Química de Sao Carlos, Universidade de Sao Paulo, Avenida Trabalhador Sãocarlense 400, 13566-590 São Carlos, SP, Brazil; § Innovative Materials and Processes for Advanced Environmental Clean Technologies (IMPACT) Research Group Laboratory, Department of Chemical Sciences, University of Padova, 35122 Padua, Italy; ∥ Chair of Urban Water Systems Engineering, Technical University of Munich (TUM), Am Coulombwall 3, 85748 Garching, Germany

## Abstract

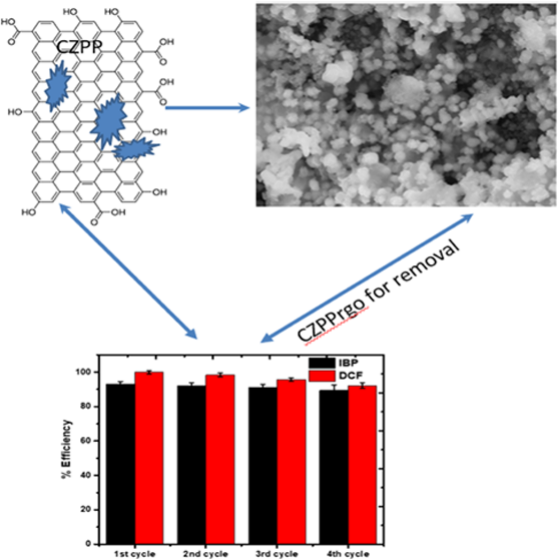

The persistent increase in the amount of nonsteroidal anti-inflammatory drugs such as ibuprofen (IBP) and diclofenac (DCF) in water bodies is alarming, thereby calling for a need to be addressed. To address this challenge, a bimetallic (copper and zinc) plantain-based adsorbent (CZPP) and reduced graphene oxide modified form (CZPPrgo) was prepared by facile synthesis for the removal of ibuprofen (IBP) and diclofenac (DCF) in water. Both the CZPP and CZPPrgo were characterized by different techniques such as Fourier transform infrared spectroscopy (FTIR), X-ray diffraction analysis (XRD), scanning electron microscopy (SEM), and pH_pzc_ analysis. FTIR and XRD confirmed the successful synthesis of the CZPP and CZPPrgo. The adsorption of the contaminants was carried out in a batch system, and several operational variables were optimized. The adsorption is affected by the initial concentration of the pollutants (5–30 mg·L^–1^), the adsorbent dose (0.05–0.20 g), and pH (2.0–12.0). The CZPPrgo has the best performance with maximum adsorption capacities of 148 and 146 mg·g^–1^ for removing IBP and DCF from water, respectively. The experimental data were fitted into different kinetic and isotherm models; the removal of IBP and DCF follows the pseudo-second order, which can be best explained by the Freundlich isotherm model. The reuse efficiency was above 80% even after four adsorption cycles. This shows that the CZPPrgo is a promising adsorbent for removing IBP and DCF in water.

## 1 Introduction

Water, a source of life and widely acknowledged as the most critical natural resource, covers most of the earth. Because of the increase in industrialization and globalization, the water bodies are polluted with contaminants such as heavy metals, dyes, pharmaceuticals, pesticides, and other contaminants such as fluoride, phenols, insecticides, pesticides, and detergents.^1^

Pharmaceuticals such as ibuprofen (IBP) and diclofenac (DCF) are a new class of pollutants identified in aquatic environments known as emerging contaminants (EC) that are on the rise and have been shown to have harmful impacts on living species.^2,3^

Also, 2-(4-(2-methylpropyl)phenyl)propanoic acid (known as IBF) and 2-(2, 6-dichloroanilino) phenylacetic acid (known as DCF) are classified as nonsteroidal anti-inflammatory drugs (NSAIDs). These classes of medication are commonly used as anti-inflammatory and antipyretic medications.^4^ IBP acts by inhabiting hormones that cause pain in the body cyclooxygenase-2 (COX-2), the dominant nonprescription pharmaceutical used worldwide.^5^ Some effects of ibuprofen are gastric ulceration, bleeding, kidney problems, bowel inflammation, mucosal damage, vomiting, and cardiovascular issues.^6^ DCF works by reducing substances that cause pain and inflammation and treating mild to moderate pain.

The current waterworks treatments were not designed to take care of these problems in the water. It necessitates the development of strategies to address the issue. Several technologies have been reportedly explored for the removal of these contaminants, such as degradation,^3,7,8^ combined advanced oxidation processes (AOPs) such as sono-photocatalytic degradation in the presence of Fe^3+^ and TiO_2_,^9^^9^ sono-enzymatic degradation,^10^ oxidation processes using ozone, enhanced ultraviolet oxidation such as UV/Fenton or photo-Fenton,^7,11,12^ hydrogen peroxide, electrodegradation,^13^ electrocoagulation,^14^ advanced oxidation technologies,^15,16^ ultrafiltration, nanofiltration, and reverse osmosis.^17^ The major shortcomings of these techniques are the high cost and production of by-products, which may be more toxic. According to a lucid analysis of all of these techniques, carbon-based adsorption generally has proven to be one of the most promising approaches for removing these contaminants from water because it is easy, cost-effective, and has zero production of toxic by-products.^18−20^

Recently, researchers have explored different materials with the potential to remove these contaminants from water, such as magnetic silica composites decorated with graphene oxide,^21^ reduced graphene oxide (RGO),^21^ and graphitic carbon nitride,^22^ to remove pharmaceutical contaminants from aqueous systems. It exhibited high adsorption capacity.^23^ Also, different soil minerals are used *via* kaolinite,^24^ montmorillonite,^25^ goethite,^26^ and activated carbon.^27^ Because of the large surface area available and the combination of well-developed pore structure and surface functional group capabilities of activated carbons, adsorption using activated carbon is suitable for removing organic molecules.^28^

Different biomasses and biomass-based materials have also been explored for the adsorption of IBP and DCF, such as cocoa shell,^29^ coconut husk,^30^ orange peel,^31^ pine sawdust,^32^ rice straw,^33^ cotton stalk,^34^ nutshell,^35^*Moringa oleifera*,^36^ potato peels,^37^*etc*. The data reported in these works have shown biomasses to be promising for the removal of contaminants. The use of cost-efficient agro-waste materials such as the listed ones reduces the high cost of adsorbent synthesis.^19^ Several means have been devised to modify the adsorbents for optimum performance; one of the widely used ones is the modification with metal oxides like iron FeO,^18,38^ CuO,^39^ ZnO,^19,40^ Al_2_O_3_,^41^ MgO,^42,43^ MnO_2_,^44^^44^ TiO_2_,^45^ etc., which improves the morphology and particle size, biocompatibility, and stability.

This current research focuses on the facile synthesis of copper- and zinc-modified biomass-based adsorbents from crushed plantain peel, reduced graphene oxide, zinc, and copper to remove DCF and IBP from water. The use of crushed plantain peel stems from its abundance in the environment and conversion of waste to wealth, and it is the activated carbon source; CuCl_2_and ZnCl_2_ were used for the modification of the plantain peel because they are affordable, easy to synthesize, and environmentally benign, and they improve the surface area, morphology, and the overall performance of the adsorbent. Reduced graphene oxide (RGO) was further used to modify the composite because of its rich π-electron system.

## 2 Materials and Methods

### 2.1 Materials

The materials used in this study were as follows: 0.22 μm poly(tetrafluoroethylene) (PTFE) membrane filters (Analitca), filter paper, cellulose nitrate filter (0.45 μm Sartorius Stedim, Germany), nylon filter (47 mm, 0.45 μm, UNIFIL), magnetic beads, graphite (Lab Synth Brazil), hydrochloric acid, potassium permanganate (Lab Synth Brazil), graphite (Lab Synth Brazil), hydrogen peroxide (Lab synth Brazil), millipore water, Extran (Lab Synth Brazil), ethanol (99.8%, Neon Brazil), copper chloride (97%, Sigma Aldrich, Germany), sodium chloride (99% Sigma Aldrich, Germany), zinc chloride (≥98%, Sigma Aldrich, Germany), centrifuge, pipette, pipette tips, IBP (≥98%, HPLC grade, Sigma Aldrich, Germany), hydrazine (98% Sigma Aldrich, Germany), and DCF (≥98%, HPLC grade, Sigma Aldrich, Germany).

### 2.2 Preparation of Adsorbents

The plantain peel was obtained from the Redeemer’s University Cafeteria, Ede, Nigeria (7°40′52″N, 4°27′29″E); they were washed with water and dried in an oven at 105 °C until weight constancy. The dried peels were obtained and ground into powder using a mortar and pestle.

The syntheses of ZnCl_2_+plantain peel (ZPP) and RGO are described in S1.1 and S1.2 of the Supporting Information (SI), respectively.

#### 2.2.1 CZPP (Zinc Chloride + Copper Chloride + Plantain Peel)

Four grams of zinc chloride, 4 g of copper chloride, and 8 g of crushed plantain peel seeds were weighed into a beaker with 10 mL of 0.1 M NaOH and stirred continuously for 20 min. This mixture was transferred into an oven at 105 °C for 24 h to allow the impregnation process. Samples from the oven-dried mixture were transferred into a furnace and heated at 500 °C at the rate of 5 °C·min^–1^ for 3 h in air. The resulting dark powdery material was washed several times with Millipore water until the pH was 7.0 to remove the unbound components from the CZPP surface. CZPP was dried in the oven at 105 °C for 6 h. After drying, the CZPP was stored in an airtight container.

#### 2.2.2 CZPPrgo (Zinc Chloride + Copper Chloride + Plantain Peel + RGO)

One gram of RGO was weighed and poured into a beaker containing methanol and water (2:1); 2 g of prepared CZPP was dispersed in a suspension containing the RGO and agitated for 30 min on a magnetic stirrer. It was then transferred to a sonicator for 3 h. The solution was decanted and dried in an oven at 105 °C for 5 h. The resulting powder was stored in an airtight container.

## 3 Characterization of the Adsorbents

The surface morphology and elemental composition of the nanocomposites were determined by a scanning electron microscope (LEO, model: 440) operated at 5 kV accelerating voltage. The scanning electron microscope was equipped with an energy-dispersive X-ray (EDX) spectrometer. Infrared spectra were collected on a PerkinElmer Spectrum 100 Fourier transform infrared (FTIR) spectrophotometer with a universal attenuated total reflectance (ATR) sampling accessory and internal reflectance element diamond. X-ray diffraction (XRD) was performed on a PANalytical Empyrean powder X-ray diffractometer in a Bragg–Brentano geometry equipped with a PIXcel1D detector using Cu Kα radiation (λ = 1.5419 Å) operating at 40 kV and 40 mA.

The method for determining the point of zero charges (pH_pzc_) is in S1.3 of the Supporting Information (SI).

### 3.1 Adsorption Study

The kinetic study was performed to determine the optimum efficiency of CZPP and CZPPrgo using 100 mL of 30 mg·L^–1^ for IBP and DCF with 0.20 g of each adsorbent. The mixtures were agitated for 480 and 120 min, respectively. At different time intervals, 2 mL aliquots were withdrawn from the sample. The concentrations of the supernatant of IBP and DCF were determined using a UV–vis spectrophotometer at 286 and 276 nm, respectively. The withdrawn samples were analyzed immediately in a span of time of less than a minute and returned into the system ruling out any perturbation like changes in concentration, volume, and temperature. The amounts of IBP and DCF removal were calculated by;
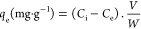
1where *q*_e_, *C*_i_, *C*_e_, *V*, and *W* are the amount of IBP and DCF (mg·L^–1^) adsorbed in mg·g^–1^, initial concentrations of the IBP and DCF (mg·L^–1^), equilibrium concentrations of IBP and DCF (mg·L^–1^), the volume of aqueous solutions (*L*), and the mass of the adsorbent (g), respectively.

#### 3.1.1 Effect of Initial Concentration

Equilibrium studies were performed at various initial concentrations using the CZPP and CZPPrgo within the range of 0–100 mg·L^–1^ in 100 mL of the analytes IBP and DCF with an adsorbent dosage of 0.2 g. The mixtures of IBP and DCF were agitated for 480 and 120 min, respectively. Then, a 2 mL aliquot was withdrawn from the sample after the reaction.

The concentration of the supernatant of IBP and DCF was determined using a UV–vis spectrophotometer at 286 and 276 nm, respectively.

The experimental data obtained were fit into some nonlinear equilibrium models, which are Freundlich (eq 2), Fritz–Schlunder (eq 3), and Elovich (eq 4), and nonlinear kinetic models, which are pseudo-second-order model (PSOM) (eq 5), fractal-like pseudo-second-order model (FL-PSOM) (eq 6), two-step kinetic model (eq 7), fractal-like kinetic model (FKN) (eq 8), and Morris–Weber intraparticle diffusion (IPD) model (eq 9).^19,46^ The experimental data were fit into the nonlinear mathematical equations below using the quasi-Newton least-squares algorithm in KyPlot Software 2.0 Model (Kyens Lab Inc., Tokyo, Japan).
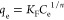
2
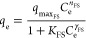
3
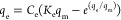
4
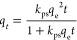
5
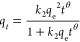
6
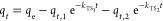
7
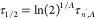
8
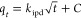
9where *k*_F_, *n*, *k*_FS_, *q*_max_FS__, γ_FS_, *K*_e_, *q*_m_, *k*_ps_, *k*_2_, θ, *k*_1_, *k*_2_^′^, α, *k*_ipd_, *C*, *n*′, *A*, *k*_1_*t*, *k*_2_^′^*t*, and τ_1/2_ are the Freundlich constant (mg·g^–1^) (L·mg^–1^)^1/*n*^, the empirical constant that represents the adsorption affinity, Fritz–Schlunder equilibrium constant (L·mg^–1^), Fritz–Schlunder equilibrium first exponent, Fritz–Schlunder equilibrium adsorption capacity (mg·g^–1^), Fritz–Schlunder equilibrium second exponent, Elovich isotherm constant, Elovich equilibrium adsorption capacity (mg·g^–1^), pseudo-second-order rate constant (g·mg^–1^·min^–1^), fractal-like pseudo-second-order exponent, fractal-like pseudo-second-order first rate constant (g·mg^–1^·min^–1^), fractal-like pseudo-second-order second rate constant, Morris–Weber intraparticle diffusion constant (mg·g^–1^)^0.5^, boundary layer exponent, fractal-like kinetic order rate constant, fractional time index, two-step kinetic first constant, two-step kinetic second constant, and time taken for 50% adsorption, respectively.

#### 3.1.2 Effect of Adsorbent Dosage

The effect of the CZPP and CZPPrgo dosage on the adsorption process was determined by varying the dosage from 0.05 to 0.2 g with IBP and DCF concentrations of 30 mg·L^–1^. The mixtures of IBP and DCF were agitated for 480 and 120 min, respectively. Then, a 2 mL aliquot was withdrawn from the sample after the reaction. The concentration of the supernatant of IBP and DCF was determined using a UV–vis spectrophotometer at 286 and 276 nm, respectively.

#### 3.1.3 Effect of pH

The effect of pH on the adsorption process of the CZPP and CZPPrgo was determined by changing the pH ranging between 2.0 and 12.0. The desired pH was adjusted with 0.01 M HCl and 0.01 M NaOH using a pH meter. The concentrations of IBP and DCF were 30 mg·L^–1^ with 0.2 g of CZPP and CZPPrgo. The mixtures were agitated for 480 and 120 min, respectively. Then, a 2 mL aliquot was withdrawn from the sample after the reaction. The concentration of the supernatant of IBP and DCF was determined using a UV–vis spectrophotometer at 286 and 276 nm, respectively.

#### 3.1.4 Effect of Anionic Interference

The effect of anions on the adsorption of IBP and DCF was studied by adding 2.0 mM HCO_3_, PO_4_^3–^, and SO_4_^2–^ into 30 mg·L^–1^ IBP and DCF solutions containing 0.20 g of the adsorbents. The mixtures were agitated for 480 and 120 min, respectively. At different time intervals, 2 mL aliquots were withdrawn from the samples. The concentration of the supernatant of IBP and DCF was determined using a UV–vis spectrophotometer at 286 and 276 nm, respectively.

#### 3.1.5 Effect of Ionic Strength

The effect of the initial ionic strength of IBP and DCF was studied by adding NaCl to 30 mg·L^–1^ IBP and DCF solutions containing 0.20 g of the adsorbent with concentrations of 0.05, 0.10, 0.15, and 0.20 mol·L^–1^. The mixtures were agitated for 480 and 120 min, respectively. At different time intervals, 2 mL aliquots were withdrawn from the samples. The concentration of the supernatant of IBP and DCF was determined using a UV–vis spectrophotometer at 286 and 276 nm, respectively.

#### 3.1.6 Reuse Efficiency

After the adsorption process had taken place, the IBP- and DCF-laden adsorbent was regenerated with ethanol, exhaustively washed with Millipore water, and then used for the adsorption of IBP and DCF again.

## 4 Results and Discussion

### 4.1 pH Point of Zero Charge

The pH at which the particle’s net charge is zero, or the number of positive and negative charges on the particle is equal, is the point of zero charges. Once this pH is reached, particles stop moving in the presence of an electric field.^47^

The value of pH_pzc_ is calculated at the point where the curve intersects the plot: pH zero line (pH versus pH_o_). The pH_pzc_ of an adsorbent is a crucial property that determines the pH at which the adsorbent surface is electrically neutral.^48^ The acidic or basic functional groups in the solution no longer contribute to the pH of the solution at the pH_pzc_ value.^19^^19^

**Figure 1:**
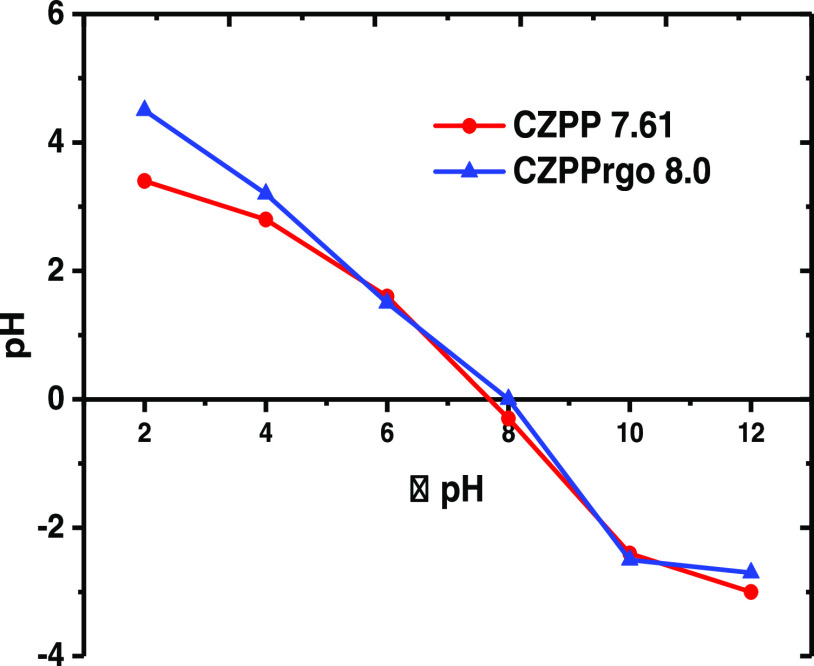
pH_pzc_ of CZPP and CZPPrgo.

The value of pH_pzc_ of the adsorbent (CZPP and CZPPrgo), as seen in Figure 1, is 7.6 and 8.0, respectively. This demonstrates that when the pH_pzc_ is exceeded, the solution becomes negatively charged, promoting the absorption of cationic species. This could be due to the activation and carbonization procedure, which eliminates volatile components and significantly dries the material.^49^ It has been observed that an ash content of 1–20% is suitable for effective pollution adsorption.

This implies that the modification of plantain peels with Cu, Zn, and RGO changed the surface characteristics of the raw plantain peel. For the adsorbents used in this study (CZPP and CZPPrgo), the adsorption process is favored at pH ≤ pH_pzc_ at 7.6 and 8.0, respectively.

### 4.2 Fourier Transform Infrared (FTIR) Spectrophotometry

**Figure 2:**
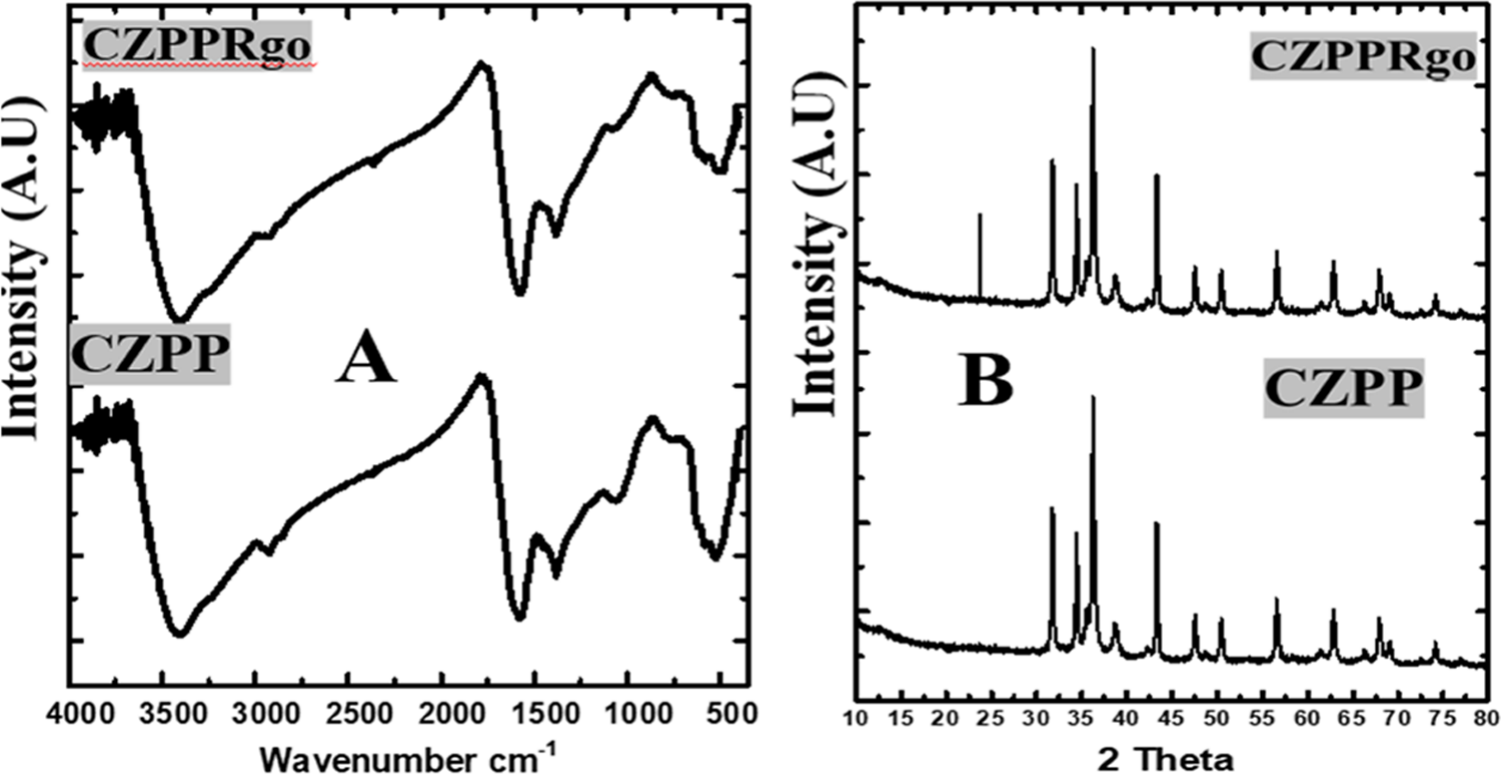
(A) Fourier transform infrared spectra and (B) X-ray diffraction spectra of CZPP and CZPPrgo.

The functional groups present in CZPP and CZPPrgo adsorbents were investigated using FTIR, as shown in Figure 2A. Notable peaks in both CZPP and CZPPrgo were observed from the results at 3400, 2971, 1609, 1381, 1071, 759, 501, 561, and 605 cm^–1^, which are hydroxyl groups of polymeric compounds such as lignin or pectin that contain the functional groups of alcohols and carboxylic acids, which can stem from the plantain peel, C–O stretch, and C–H stretch, respectively, N–H bending vibrations of primary amines, ZnO^19^ and CuO^49,50^ respectively.

The O–H signal at 2971 cm^–1^ appeared to be of low intensity, probably caused by the intense moisture loss resulting from modification of the biomass during its synthesis. In CZPPrgo, a considerable drop in the intensity of all oxygen-containing moieties was observed, implying that graphene oxide is efficiently converted to reduced graphene oxide. All of this confirms the successful incorporation of all of the components.

### 4.3 X-ray Diffraction

The formation of new phases was confirmed using the XRD tool; both the adsorbents CZPP and CZPPrgo had similar peaks at 31.6, 34.5, 47.6, 56.7, 63.2, and 68.1°, as shown in Figure 2B, which confirms the zincite phase of the zinc oxide, resulting from the doping the plantain peel with zinc chloride and sodium hydroxide (JCPDS 05-0664).
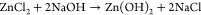


Upon calcination, the zinc hydroxide is converted to zinc oxide (zincite phase).
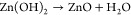


The peaks at 38.9, 61.1, 63.0, and 66.3° confirm the tenorite phase of the copper oxide, resulting from the addition of copper chloride and sodium hydroxide to the plantain peel; the reaction leads to the formation of copper oxide (JCPDS 048-1548).
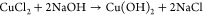


Upon calcination of the adsorbents, the copper hydroxide present produces copper oxide (tenorite).
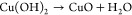


The cupric phase (Cu_2_O) was observed at 43.4 and 76.4°, which indicates the reduction of the Cu(II) species to Cu(I) species (JCPDS 05-0667). The CZPPrgo showed a peak at 23.7°, corresponding to the (002) lattice plane attributed to the reduced graphene oxide in the adsorbent because some of the functionalities have been reduced.^51^ Results from both FTIR and XRD analyses confirmed the successful incorporation of the different components of zinc, copper, and reduced graphene oxide together in the adsorbent.

### 4.4 Scanning Electron Microscopy

**Figure 3:**
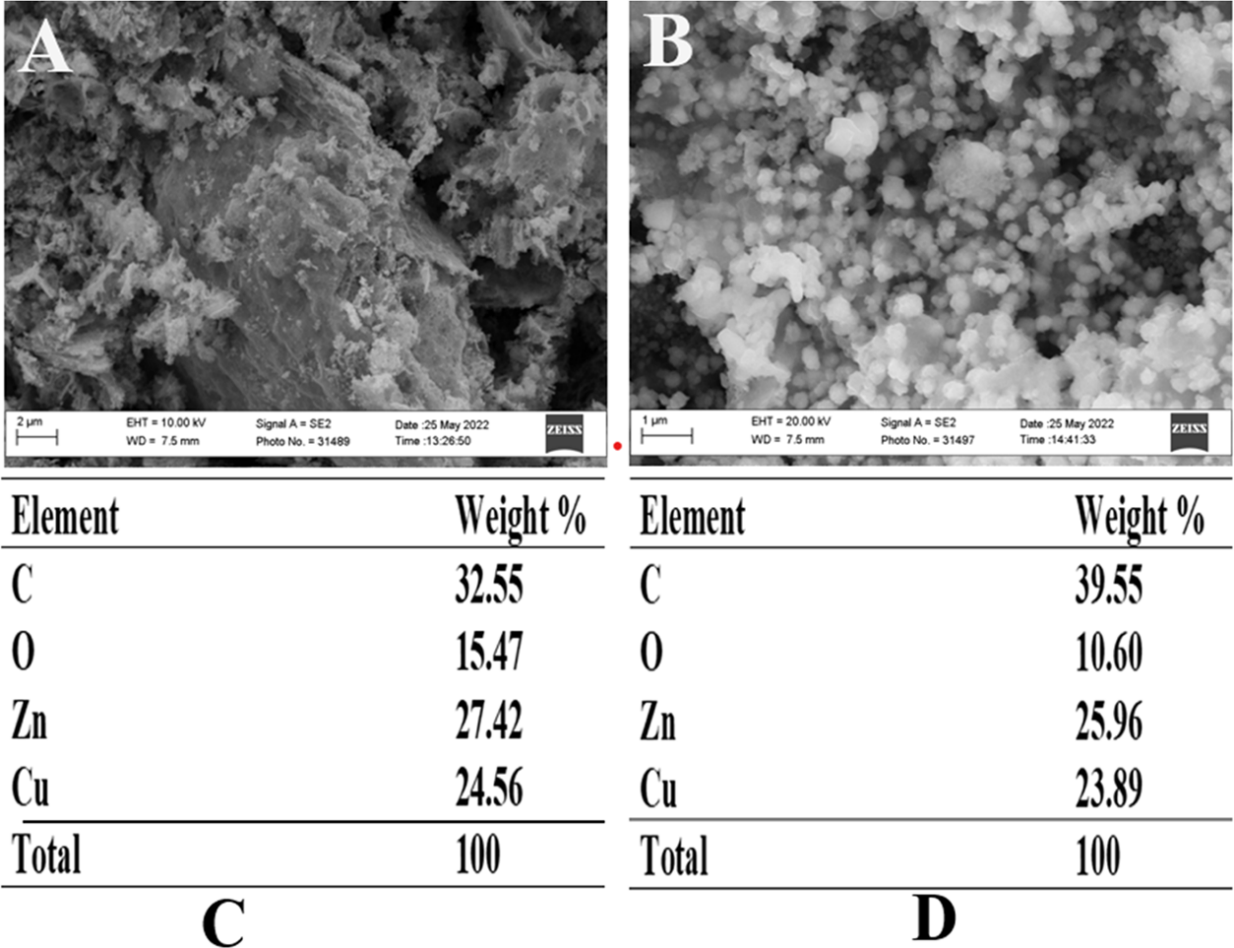
Scanning electron microscopy images of (A) CZPP and (B) CZPPrgo and energy-dispersive X-ray spectroscopy table of (C) CZPP and (D) CZPPrgo.

The surface morphologies of CZPP and CZPPrgo were examined using a scanning electron microscope. The image of the CZPP showed particles of different oval shapes and sizes closely packed together, which can be because of the zincite and tenorite phases, as confirmed by the XRD result, as shown in Figure 3. The CZPPrgo showed relatively homogeneous particles that were evenly distributed on the surface of the adsorbent. Also, a sheet-like morphology was observed on the surface of the adsorbent, which stems from the reduced graphene oxide.

### 4.5 Preliminary Studies

To understand the influence of each component of the CZPP and CZPPrgo composite on the removal of IBP and DCF from water, experiments were performed using the method described in Section 3.1. Also, the samples were withdrawn after 480 and 120 min, respectively.

**Figure 4:**
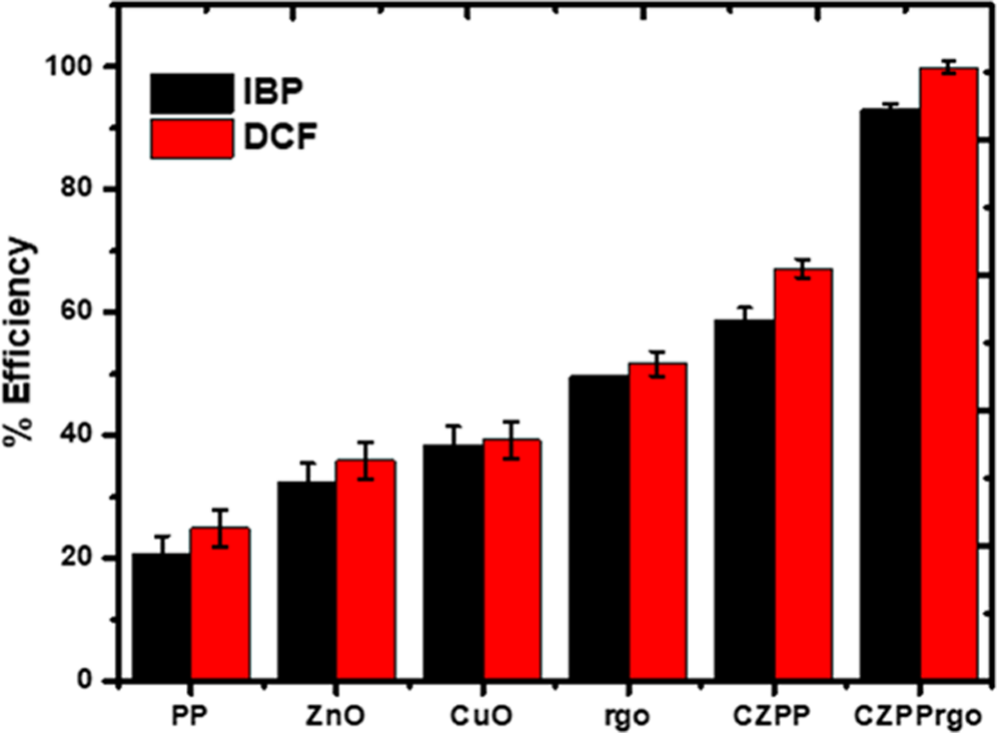
Efficiency of the different components: plantain peel, zinc oxide, copper oxide, RGO, and the composite CZPP and CZPPrgo for the removal of IBP and DCF in water.

Figure 4 shows that the PP alone showed a removal of >20%, CuO and ZnO showed a removal of >40%, and RGO exhibited a removal of >50%. Upon modification of the PP with copper oxide and zinc oxide (CZPP), the efficiency improved, and it displayed a removal of >80%. Further modification with RGO improved the adsorbent CZPPrgo performance with over 90% removal of both IBP and DCF, respectively.

### 4.6 Adsorption Kinetics

The effect of contact time for the adsorption of Ibuprofen and diclofenac onto two adsorbents CZPP and CZPPrgo was investigated between 5–480 and 5–120 min, respectively.

To understand the adsorption process, four nonlinear kinetic models were utilized in the kinetic experimental data analysis, which are the pseudo-second-order model (PSOM), fractal-like pseudo-second-order model (FL-PSOM), fractal kinetic model (FKN), two-step kinetic models, and intraparticle diffusion model (IPD).

**Figure 5:**
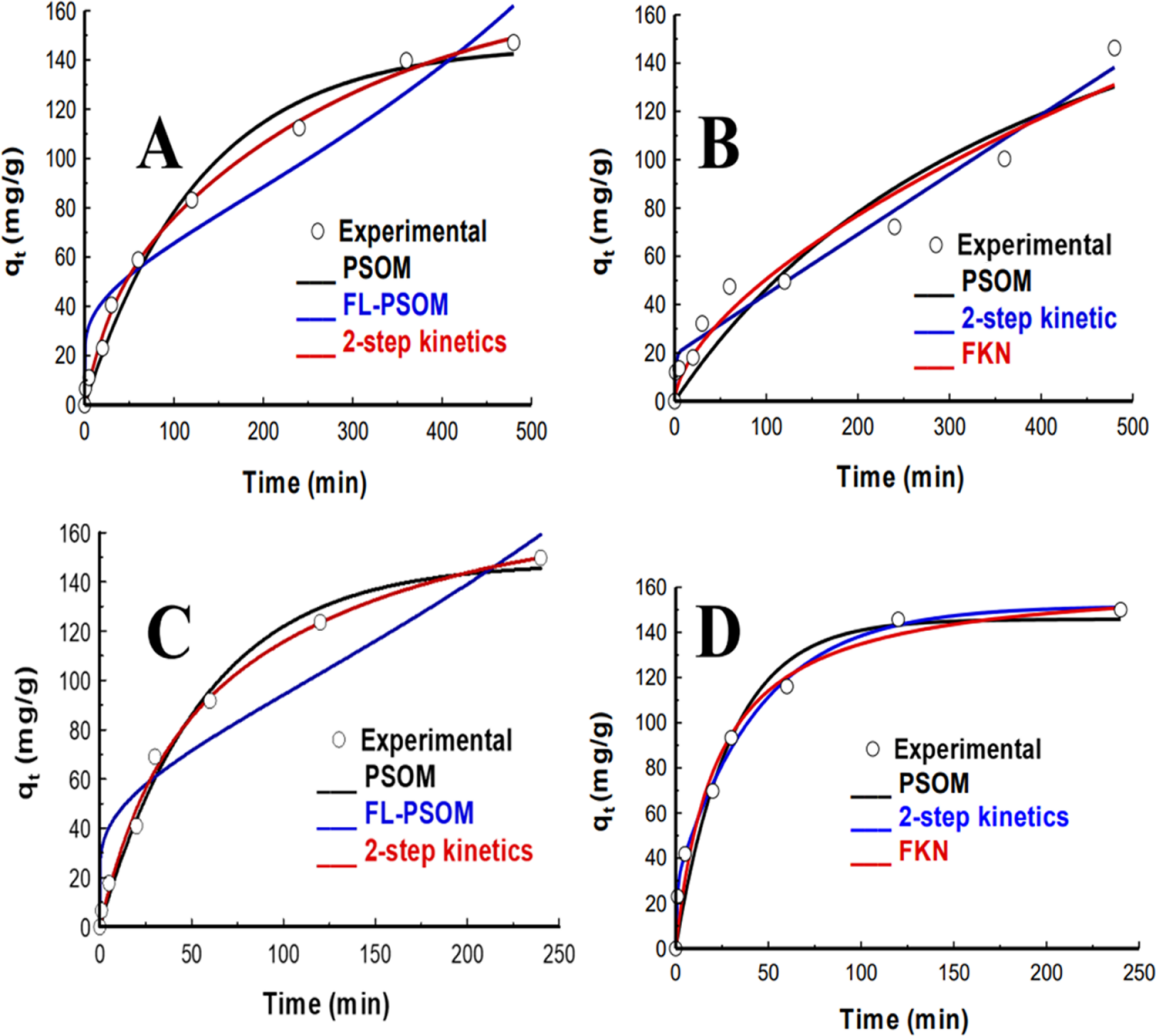
Kinetics model fittings of (A) IBP, (B) DCF adsorption by CZPP, (C) IBP, and (D) DCF adsorption by CZPPrgo.

The kinetic parameter data of all of the fitted models and *R*^2^ value determined by nonlinear regression analysis are reported in Figure 5A–D and Table 1 below. To quantitatively compare the accuracy of the models in describing the experimental data obtained, the correlation coefficient was used.

**Table 1:** Adsorption Kinetics Parameters of IBP and DCF on CZPP and CZPPrgo for Some Models

Kinetics	IBP	IBP	Kinetics	DCF	DCF
	CZPP	CZPPrgo		CZPP	CZPPrgo
PSOM			PSOM		
*q*_e_ (mg g^–1^)	136.1552	147.7790	*q*_e_ (mg g^–1^)	248.8234	145.7965
*K*_ps_ (min^–1^)	0.0077	0.0174	*K*_ps_ (min^–1^)	9.2 × 10^–6^	0.0339
*R*^2^	0.9868	0.9908	*R*^2^	0.9985	0.9806
FL-PSO			FKN		
*q*_e_ (mg g^–1^)	36.9783	24.4841	*n^i^*	142.1205	163.0799
*K*_2_ (g mg^–1^ min^–1^)	0.1962	0.2607	*t*^1/2^ (min^–1^)	1.0650	0.0005
θ	0.0437	0.0234	*A*	34.8633	39.8873
*R*^2^	0.9635	0.97028	*R*^2^	0.9773	0.9760
Two-step kinetics			Two-step kinetics		
*q*_e_ (mg g^–1^)	128.9782	110.0241	*q*_e_ (mg g^–1^)	168.5467	123.0799
*K*_1_ (min^–1^)	0.0036	0.0080	*K*_1_ (min^–1^)	0.3730	0.0223
*K*_2_^i^ (min^–1^)	0.0377	0.0414	*K*_2_^i^ (min^–1^)	0.7488	1.2410
α	34.6273	24.9458	α	19.2803	28.5981
*R*^2^	0.9867	0.9753	*R*^2^	0.9752	0.9976
IPD			IPD		
*K*_ipd_ (mg g^–1^)^1/2^	7.0973	5.6532	*K*_ipd_ (mg g^–1^)^1/2^	10.4130	10.0817
*C*	0.7862	0.6274	*C*	0.3245	0.2854
*R*^2^	0.9894	0.9443	*R*^2^	0.9899	0.9068

The *R*^2^ value of the model was taken into consideration, and based on the high *R*^2^ values obtained, it was observed that the experimental data of CZPP and CZPPrgo for the adsorption of IBP and DCF best fitted the PSO model. It assumes that a biomolecular interaction involving ion exchange between the adsorbent and the adsorbate is the rate-limiting step responsible for the adsorption of IBP and DCF.^52^ The pseudo-second-order model is generally applied to heterogeneous materials;^53^ the adsorption of IBP and DCF onto the surface of the adsorbents CZPP and CZPPrgo is suspected to be through ionic interaction with the OH groups on the surface of the adsorbents.^54^ This depicts that chemisorption ruled the adsorption while the active site on the adsorbent surface determined the adsorption capacity. The *q*_e_ value of the PSOM for the adsorption of both contaminant IBP and DCF by CZPP and CZPPrgo was higher than those of the other kinetic models, further confirming it as the best fit for the analysis.

**Figure 6:**
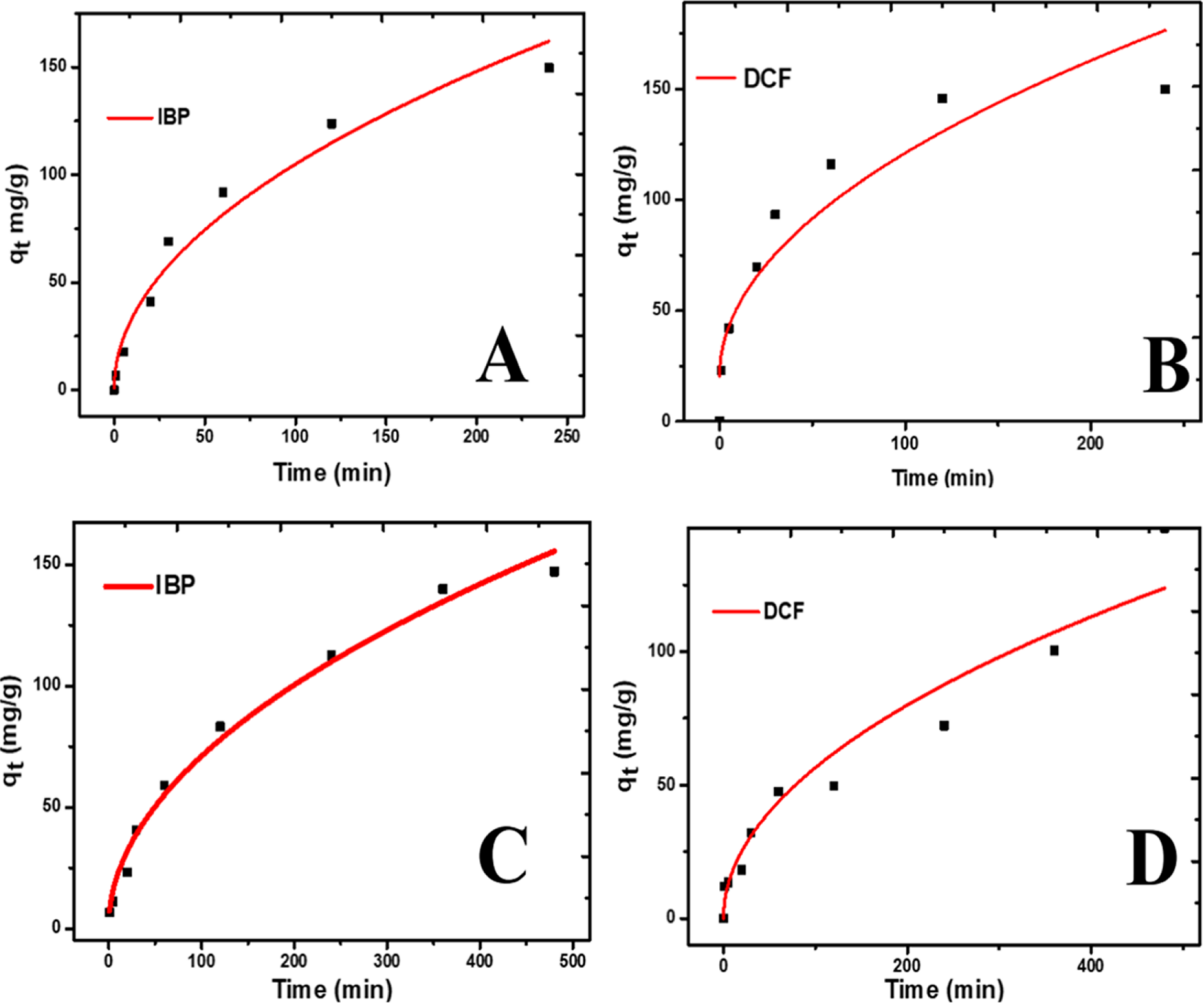
Intraparticle diffusion kinetics model fittings of (A) IBP, (B) DCF adsorption by CZPP, (C) IBP, and (D) DCF adsorption by CZPPrgo.

Intraparticle diffusion (IPD) (Figure 6A–D) suggests that if the plots *q_t_* against √*t* is linear and passes through the origin, the sole rate-limiting step is the intraparticle diffusion, but otherwise, it implies that other processes are involved in the mechanism of adsorption.^55^ The curve fitting of the IPD model for the adsorption of IBP and DCF passed through the origin suggests that the primary process is the IPD. The value of *C* indicates the effect of the boundary layer on the adsorption process from the *C* values,^56^ as reported in Table 1; it shows a more significant *C* value for CZPP as compared to CZPPrgo, which implies that the boundary layer affected the adsorption process in CZPP and less impact on the CZPPrgo, which can be confirmed from the adsorption performance of the CZPPrgo.

### 4.7 Adsorption Isotherms

**Figure 7:**
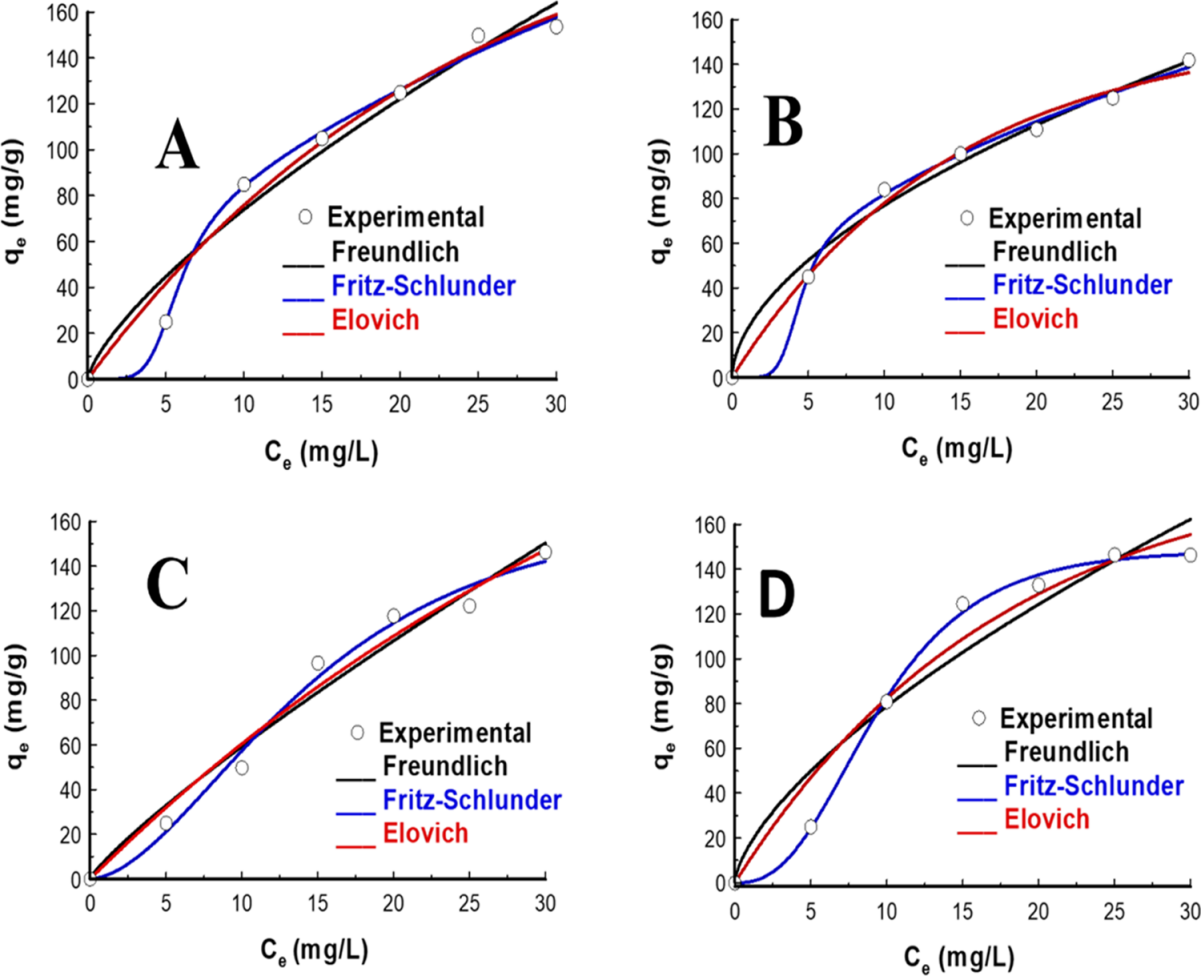
Adsorption isotherm model fits of (A) IBP, (B) DCF adsorption by CZPP, (C) IBP, and (D) DCF adsorption by CZPPrgo.

The adsorption isotherms (Figure 7A–D) were used to estimate the amount of contaminants adsorbed on the adsorbent (CZPP and CZPPrgo) at equilibrium. The experimental data generated were fitted into three equilibrium models: Freundlich, Fritz–Schlunder, and Elovich models.

The Freundlich isotherm model, a two-parameter isotherm, best fitted the experimental data of the adsorption of both IBP and DCF using CZPP and CZPPrgo, followed by the Fritz–Schlunder and Elovich models.

The implication of the adsorption isotherm data best fitting the Freundlich is that the adsorption of IBP ad DCF was through multilayer heterogeneous adsorption. The 1/*n* value for both IBP and DCF using CZPP and CZPPrgo was >1, as shown in Table 2, indicating that the adsorption was unfavorable. This suggests that the isotherm is linear and is of type C.^57^ The model exponents of the Fritz–Schlunder isotherm, η_fs_ and γ_fs_, for both IBP and DCF are very far from unity; this indicates that the adsorption data did not follow the hypotheses of the Langmuir isotherm.^57^ This also suggests that it has been reduced to the Freundlich model.

**Table 2:** Adsorption Isotherm Parameters of IBP and DCF on CZPP and CZPPrgo for Various Models

isotherm	IBP	DCF		IBP	DCF
	CZPP		CZPPrgo		
Freundlich			Freundlich		
*K*_F_ (mg g^–1^) (L mg^–1^)^1/*n*^	8.4201	17.3205	*K*_F_ (mg g^–1^) (L mg^–1^)^1/*n*^	13.8904	21.4548
*n*	1.1801	1.5202	*n*	1.3776	1.8041
*R*^2^	0.9722	0.9740	*R*^2^	0.9677	0.9973
Fitz–Schlunder			Fitz–Schlunder		
*q*_maxFS_ (mg g^–1^)	18.8812	42.2589	*q*_maxFS_ (mg g^–1^)	6.0408	6.5537
*K*_FS_ (L mg^–1^)	1.4375	2.5934	*K*_FS_ (L mg^–1^)	0.0026	0.0049
η_FS_	11.7223	25.5074		5.4941	6.0789
γ_FS_	0.0043	0.1080	γ_FS_	0.0001	0.0002
*R*^2^	0.9879	0.9979	*R*^2^	0.9965	0.9978
Elovich			Elovich		
*K*_e_ (L g^–1^)	0.02226	0.5665	*K*_e_ (L g^–1^)	0.0415	0.0690
*q*_m_ (mg g^–1^)	298.6145	190.2173	*q*_m_ (mg g^–1^)	223.2549	155.5305
*R*^2^	0.9778	0.96165	*R*^2^	0.9799	0.9921

### 4.8 Optimization of Operational Variables

#### 4.8.1 Effect of Adsorbent Dose

**Figure 8:**
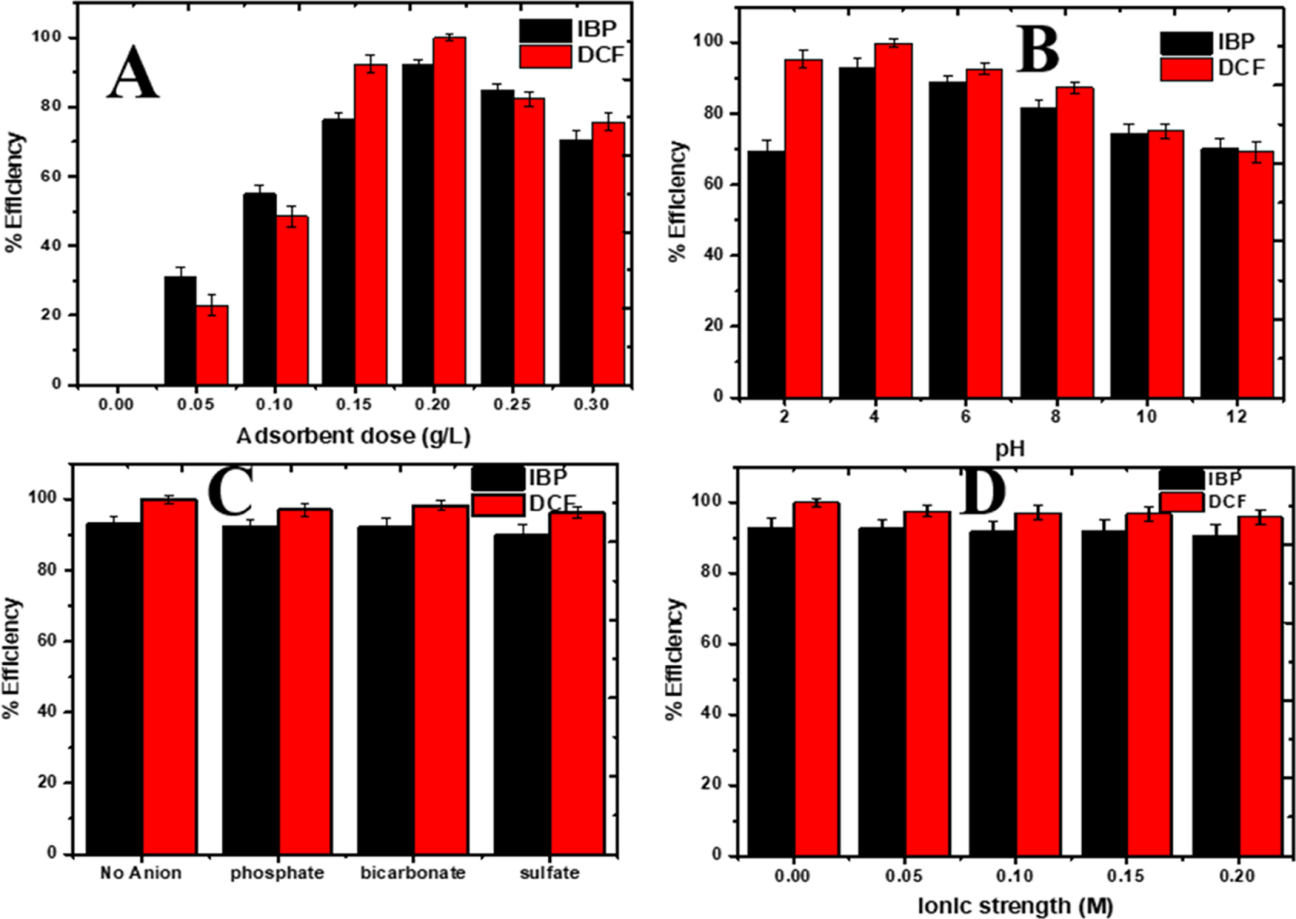
Effect of (A) adsorbent dose, (B) pH, (C) anionic interference, and (D) ionic strength on the adsorption of IBP and DCF onto CZPPrgo.

To explore the effect of the adsorbent amount on removal efficiency and avoid the use of excess adsorbent leading to waste, different concentrations of CZPPrgo adsorbent of 0.005–0.20 g·L^–1^ were studied using constant IBP and DCF concentration (15 mg·L^–1^). It was observed that IBP and DCF increased as the adsorbent was increased from 0.05 to 0.20 g·L^–1,^ with IBP and DCF showing 92.95 and 99.91%, respectively (see Figure 8A). Increasing the adsorbent dose has different conflicting effects on the adsorption system. The increase in the adsorbent dose leads to a rise in the number of available active sites to enhance the adsorption activity and increase the removal rate.^19^ Generally, the decline in IBP and DCF removal with increasing dosage of CZPPrgo may be ascribed to the agglomeration of the nanoparticles, which leads to a smaller surface area, thereby lowering the adsorption process due to a poor adsorbent–adsorbate interaction.^58^

#### 4.8.2 Effect of pH

The effect of pH on the removal of contaminants IBP and DCF was evaluated because pH plays a vital role in the adsorption of organic contaminants from water because it could affect the charges on the surface of the contaminant.

The optimum efficiency of the CZPPrgo for removing IBP and DCF was observed at a lower pH range. The pH_pzc_ could be used to explain the effect of pH on the adsorption process. The pH_pzc_ of CZPPrgo is 8.0 with the maximum adsorption at pH 4.0. At pH > pH_pzc_, IBP is deprotonated, and a negative charge develops on the carboxyl group present in the CZPPrgo, leading to a strong dipole–dipole interaction between the CZPPrgo and IBP.

The adsorption efficiency at a lower pH could be because of the π–π stack interaction between the phenyl ring π-electrons and the CZPPrgo π-electrons. Also, the adsorption process of IBP using CZPPrgo could be by trapping the IBP molecules in the pores of the adsorbent, and this process is very dependent on pH. At a pH value lower than that of the PZC, IBP will be in a deprotonated state, making the adsorption process very easy because, at this state, the IBP becomes very hydrophobic. For the DCF, the adsorption process was favored at pH 2.0 and 4.0. DCF is considered a weak acid as its pK_a_ is around 4.15.^59^

The DCF is the most hydrophobic species; hence, some interactions between it and the CZPPrgo can impact adsorption. The pH_pzc_ of CZPPrgo is 8.0, and the pH value with the optimal removal of DCF was between 3.0 and 4.0, which is lower than the PZC in the acidic region (see Figure 8B). The plausible adsorption mechanism could be based on electrostatic interactions, H-bonding, hydrophobic effects, and π–π stack interactions. Oxygen-containing functional groups, such as carboxylic acids in the adsorbents, facilitated the adsorption through H-bonding irrespective of pH.^60^ Also, hydroxyl and amine groups, which are polar functional groups, have an electron-withdrawing effect at basic pH and can cause these groups to interact with aromatic rings, which are the π-electron acceptors in the adsorbent CZPPrgo.^61^

#### 4.8.3 Effect of Anionic Interference

As shown in Figure 8C, of the three inorganic anions used, sulfate had an effect on the adsorption efficiency of the CZPPrgo because it will compete with the contaminants IBP and DCF for the available active sites. Phosphate and bicarbonate did not affect the adsorption efficiency.

#### 4.8.4 Effect of Ionic Strength

The effect of ionic strength on the adsorption efficiency of the CZPPrgo for the removal of IBP and DCF was evaluated because, in actual life samples, the presence of ions cannot be ruled out.

The study shown in Figure 8D showed that the presence of NaCl does not really affect the adsorption efficiency of the adsorbent. This can be due to the salting out effect; it has been reported that the addition of salt to the system can increase the hydrophobicity of the microcontaminants. Looking at the pK_α_ value of both IBP and DCF, it is higher than 4, and they are considered to be hydrophobic, making them easily adsorbed to the adsorbent.^62^

#### 4.8.5 Reuse Efficiency

In addition to the excellent adsorption performance of an adsorbent, other factors like reusability and multicycle utilization are essential and crucial. Ethanol with a high dipole moment was used as the desorption reagent to regenerate the CZPPrgo. First, the IBP and DCF particles were adsorbed onto the adsorbent CZPPrgo. After the adsorption process, the IBP- and DCF-loaded adsorbent was regenerated with ethanol, exhaustively washed with millipore water, and then reused for further adsorption of IBP and DCF. The CZPPrgo was reused four times to check its stability and sustainability.

**Figure 9:**
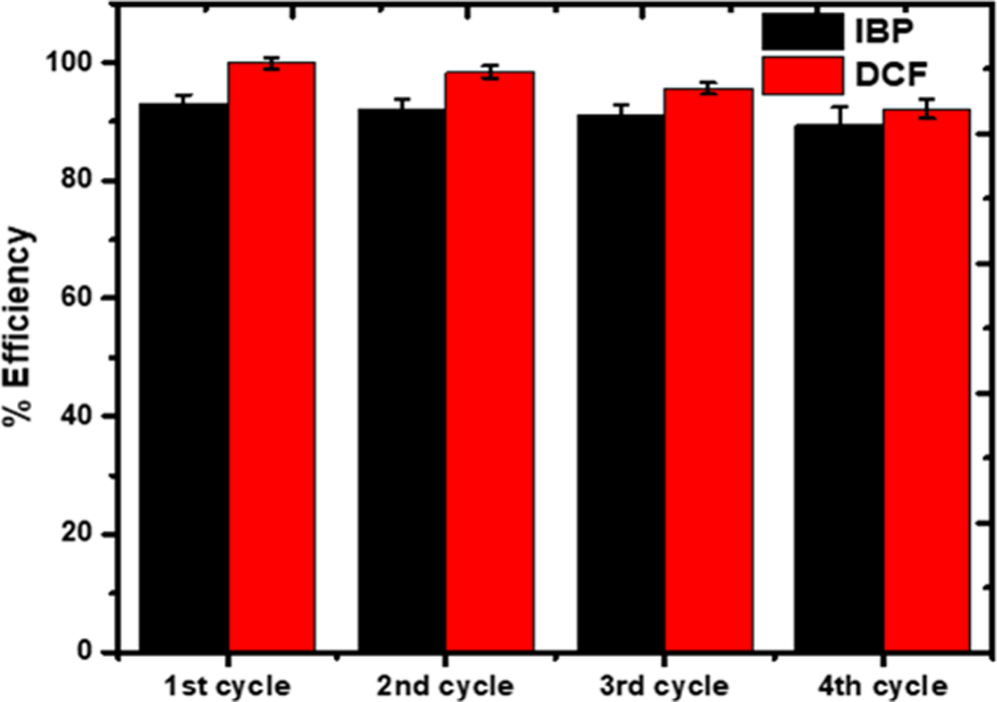
Reuse of CZPPrgo over four cycles to remove IBP and DCF in water.

The efficiency of the adsorbent CZPPrgo was evaluated over four different cycles for the removal of IBP and DCF; it showed no significant loss of efficiency over the four cycles, as demonstrated in Figure 9. The adsorption capacity of CZPPrgo used in this study was compared with other adsorbents used for the removal of IBP and DCF from water at their optimum performance.

The adsorbent used, target contaminant, and adsorption capacity are listed. The adsorption capacity of CZPPrgo is higher than some of the reported adsorbents, confirming that the CZPPrgo is an excellent adsorbent with good stability for the removal of the IBP and DCF in water (Table 3).

**Table 3:** Performance Comparison of Different Adsorbents for the Removal of IBP and DCF in Water

Adsorbents	Contaminants	Adsorption capacity (mg·g^–1^)	References
Bare CNC aerogel	DCF	11.5	(63)
CNC-PVAm/rGO		606	
Aerogel			
RGO	DCF	59.7	(64)
Porous carbons derived from MOF (PCDM-1000)	IBP	320	(65)
	DCF	400	
CTAB-modified bentonite (2CECRB)	IBP	601	(66)
	DCF	195	
Raw cocoa shell	IBP	23.8	(29)
H_2_SO_4_-OS	IBP	49.3	(67)
	DCF	73.5	
Alginate/carbon films	DCF	29.9	(68)
(AC/films)			
Chitosan-modified waste tire crumb rubber	IBP	70.0	(69)
	DCF	17.7	
	Naproxen	2.3	
Fe_3_O_4_-FeBTC MOF	IBP	238	(70)
	DCF	69.4	
	Naproxen	115	
MIL-53(AL)	DCF	297	(71)
	Naproxen	422	
Three-dimensional reduced graphene oxide aerogel (rGOA)	DCF	597	(72)
CZPP	IBP	136	This study
	DCF	249	
CZPPrgo	IBP	148	This study
	DCF	146	

## 5 Conclusions

CZPP and CZPPrgo were synthesized by a facile route. The synthesized adsorbents were characterized using FTIR, XRD, and SEM. The FTIR showed peaks that suggested the presence of ZnO and CuO and was further confirmed by the XRD analysis, indicating the successful preparation of the adsorbents. The surface area and porosity characterization also give essential information about the material, which we will include in our subsequent study. The as-synthesized adsorbents exhibit good adsorption affinity for the removal of IBP and DCF with the performance with the maximum adsorption capacity of 147.78 and 145.79 mg·g^–1^, respectively.

Different experimental variables were optimized, and they showed that the optimum removal of both IBP and DCF was obtained at an initial concentration of 30 mg·L^–1^, pH of 4.0, and adsorbent dosage of 0.2 g. The experimental data were fitted into different kinetic and isotherm models; the removal of IBP and DCF follows the pseudo-second order, which can be best explained by the Freundlich isotherm model. The adsorption mechanism could be based on electrostatic interactions, H-bonding, hydrophobic effects, and π–π stack interactions with reuse efficiencies of over 80% even after four cycles. The use of the CZPPrgo for removing the two nonsteroidal anti-inflammatory drugs, IBP and DCF, has proven effective and sustainable.

## Supporting Information Available

ZPP (ZnCl_2_ + plantain peel) (S1.1); synthesis of reduced graphene oxide (S1.2); and determination of the point of zero charge (pH_pzc_) (S1.3) (PDF)
