# Supplementary material for: Biomass-Tuned Reduced Graphene Oxide@Zn/Cu: Benign Materials for the Cleanup of Selected Nonsteroidal Anti-inflammatory Drugs in Water
Source: ACS Omega. 2023 Feb 14;8(8):7956–67. doi: 10.1021/acsomega.2c07769 (PMC9979318; doi:10.1021/acsomega.2c07769)
Supplement: Supplementary file 1 — ao2c07769_si_001.pdf [file ao2c07769_si_001.pdf]

Biomass-tuned reduced graphene oxide@Zn/Cu: Benign materials for the cleanup of selected non-steroidal anti-inflammatory drugs in water

Ajibola A. Bayode[1,2,3], Mercy T. Folorunso[1], Brigitte Helmreich[4], *Martins O. Omorogie[1,4]

[1]Department of Chemical Sciences, Faculty of Natural Sciences, Redeemer's University, P.M.B. 230, 232101, Ede, Nigeria

[2]Laboratório de Química Analítica Ambiental e Ecotoxicologia (LaQuAAE), Departamento de Química e Física Molecular, Instituto de Química de Sao Carlos, Universidade de Sao Paulo, Avenida Trabalhador Sãocarlense 400, São Carlos–SP 13566-590, Brazil

[3]Innovative Materials and Processes for Advanced Environmental Clean Technologies (IMPACT) Research Group Laboratory, Department of Chemical Sciences, University of Padova, Padua, Italy

[4]Chair of Urban Water Systems Engineering, Technical University of Munich (TUM), Am Coulombwall 3, 85748, Garching, Germany

*Corresponding Author: omorogiem@run.edu.ng, mo.omorogie@tum.de

Supporting Information File (SIF)

S1.1 ZPP ($ZnCl_2$+ plantain peel)

The ZPP was prepared according to the procedure reported in our previous study. Four grammes of Zinc Chloride and 4 g of crushed plantain peel were weighed into a beaker of 0.1 M NaOH and stirred continuously for 20 min. This mixture was transferred into an oven at 105 °C for 24 h for impregnation to take place. Samples from the oven-dried mixture were transferred into the furnace and heated at 500 ^{o}C at the rate of 5°C/min for 3 h in air. The resulting dark powdery material was washed several times with Millipore water until the pH is 7.0, ZPP was dried in the oven at 105 °C for 6 h. After drying, the ZPP was stored in an airtight container.

S.1.2 Synthesis of Reduced Graphene Oxide (RGO)

The graphene oxide was prepared by the Hummers method. Three grammes[1] of the prepared graphene oxide was weighed in a beaker; 20 mL of water was added and ultra-sonicated for 3 h. After, 3 µL of hydrazine was introduced into the solution and allow shaking for 24 h; the product was filtered and washed with Millipore water to remove some of the unbounded hydrazine.

S1.3 Determination of Point of Zero Charge (pHpzc)

The salt addition method was adopted for pHpzc determination as described in our previous study[2].

References

(1) Cao, N.; Zhang, Y. Study of reduced graphene oxide preparation by Hummers' method and related characterization. *Journal of Nanomaterials* **2015**, *2015*.

(2) Bayode, A. A.; Agunbiade, F. O.; Omorogie, M. O.; Moodley, R.; Bodede, O.; Unuabonah, E. I. Clean technology for synchronous sequestration of charged organic micro-pollutant onto microwave-assisted hybrid clay materials. *Environmental Science and Pollution Research* **2020**, *27*, 9957-9969.
